# Evaluation of Litchi Honey Quality in Southern China

**DOI:** 10.3390/foods14030510

**Published:** 2025-02-05

**Authors:** Cuiping Zhang, Shujing Zhou, Chenxinzi Wu, Xinjian Xu, Xiangjie Zhu

**Affiliations:** 1 College of Animal Sciences, Zhejiang University, Hangzhou 310058, China; lgzcplyx@zju.edu.cn; 2 College of Bee Science and Biomedicine, Fujian Agriculture and Forestry University, Fuzhou 350002, China; sjzhou118@126.com (S.Z.); wuchenxinzi@163.com (C.W.); 3 Honeybee Research Institute, Fujian Agriculture and Forestry University, Fuzhou 350002, China

**Keywords:** litchi (*Litchi chinensis* Sonn.), honey, carbohydrate, amylase value, conductivity, isotope detection, HMF, volatile components, CAC, HS-SPME-GC-QQQ

## Abstract

Honey is a sweet substance laboriously collected and crafted from nectar by bees, and since ancient times, it has been deeply cherished by humans for its unique flavor and nutritional value. Litchi honey stands out among various types of honey with its unique flavor and sweet taste, and it is particularly favored by consumers. In accordance with the testing methodologies specified in relevant Chinese national standards, we conducted an exhaustive analysis of the physicochemical properties of six litchi honey samples in Southern China. The results showed that the moisture content fell within a range of 17.18% to 22.7%, while the electrical conductivity remained below 0.28 mS/cm, and amylase activity surpassed 7.7 mL/(g·h). The fructose content varied from 36.5% to 39.6%, with glucose content ranging between 30.57% and 37.63%. The combined total of these two monosaccharides was found to be within the spectrum of 69.63% to 77.23%, and sucrose content was recorded between 0.59% and 1.15%. The F/G was between 1.05 and 1.28, the proportion of fructose in reducing sugars ranged from 51.28% to 56.22%, and the maltose content was between 1.09% and 1.51%. The HMF content was measured between 1.04 and 3.49 mg/kg. Moreover, the presence of C-4 plant sugars was absent in all tested honey samples. These results definitively demonstrate that the physicochemical attributes of all litchi honey samples align with the standards set forth by Chinese national regulations and international authorities such as CODEX. During our in-depth examination of volatile constituents, we identified 26 common compounds, with trans-linalool oxide, linalool, lilac aldehyde B, lilac aldehyde D, α-terpineol, and cedrol emerging as pivotal in crafting the unique flavor and aroma profile of litchi honey. Additionally, the detection of methyl cyclosiloxane in litchi honey has garnered our attention, necessitating a comprehensive investigation into the honey production process. In conclusion, this study not only establishes a robust scientific basis for the quality assurance and product development of litchi honey but also provides valuable reference information for consumers in their selection of honey products.

## 1. Introduction

For thousands of years, honey has been widely recognized for its high nutritional value and medicinal properties. Honey is the natural sweet substance produced by honey bees from the nectar of plants or from secretions of living parts of plants or excretions of plant-sucking insects on the living parts of plants, which the bees collect, transform by combining with specific substances of their own, deposit, dehydrate, store and leave in the honeycomb to ripen and mature by Codex Alimentarius Commission (CAC). It consists of a variety of components: 70% saccharides, with fructose and glucose being the main sugars, and 10% water, as well as organic acids, mineral salts, vitamins, proteins, and phenolic compounds. The multifunctionality of honey has gained widespread recognition in the scientific community. It is renowned not only as a natural sweetener but also as a functional food, possessing significant antioxidant, antimicrobial, antibacterial, and bacteriostatic properties. Additionally, honey is used as an antiseptic, has prebiotic and probiotic effects, is capable of modulating the immune system, and possesses multiple benefits, including anti-inflammatory, anti-tumor, and anti-cancer properties [1].

Litchi (*Litchi chinensis* Sonn.) honey, also known as lychee honey, is a unique monofloral honey primarily made from the sweet nectar of litchi blossoms. Litchi, as a delectable tropical fruit characteristic of South China, is also one of the precious fruit trees, with relatively little cultivation in other parts of the world, whereas China boasts large-scale commercial cultivation. Guangdong, Fujian, Guangxi, and Hainan are the main litchi-producing regions in our country, which include well-known varieties such as Nuomizhi, Guiwei, and Feizixiao [2,3]. Since ancient times, litchi has been widely regarded as a functional food used in the treatment of various diseases [4]. The litchi orchards in these regions provide a rich source of nectar for bees, resulting in the production of this distinctive and highly sought after honey variety. Litchi honey is characterized by its unique taste and aroma, which closely resemble the fragrance of litchi flowers. It has a light amber color and a smooth, velvety texture [5]. Due to its distinctive characteristics, litchi honey is highly valued in the market. However, its quality can vary, and it is essential to ensure the purity of the honey to maintain its monofloral status.

The production of litchi honey is subject to a multitude of factors, such as the variety of litchi flowers, the geographical placement of the hives, the specific species of bees, and the climatic conditions prevalent during the flowering season. The analysis of physicochemical parameters and the identification of volatile compounds in litchi honey are important methods for evaluating honey quality and origin [5,6,7]. Parameters such as moisture content, electrical conductivity, and color can reflect the physical state and processing of honey, while amylase and sugar are closely related to nutritional value and taste [8,9,10]. The analysis of volatile compounds is crucial for revealing the aroma characteristics and plant origin of honey. Using GC/MS fingerprinting, specific volatile compounds can be identified, which serve as markers to help distinguish honey from different sources [11,12,13,14]. Wang et al. [15] utilized headspace solid-phase microextraction in tandem with gas chromatography-mass spectrometry(HS-SPME-GC/MS) to conduct an exhaustive analysis of 30 litchi honey samples. The study found that the volatile components of litchi honey from different regions were surprisingly consistent, successfully identifying 41 unique volatile compounds and constructing a fingerprint spectrum containing 16 common characteristic peaks. Zhu et al. [16] discovered that 3,5,5-trimethoxybenzaldehyde was a characteristic component of litchi honey. Furthermore, Wang et al. [17] Utilized the HS-SPME-GC/MS to identify 98 volatile compounds from litchi honey of various origins, mainly including terpenes, alcohols, aldehydes, and aromatic compounds, among which the content of oxidized linalool was particularly prominent.

Codex standards are established by CAC with the aim of protecting consumer health, promoting fair trade, and driving food safety. The national standards for honey in China draw extensively from CAC, demonstrating the country’s commitment to and alignment with international food safety standards. Litchi honey, a rare variety of honey in South China, possesses unique quality and aroma characteristics. This paper conducted detailed measurements of the physicochemical properties of litchi honey using the methods prescribed by current national and industrial standards in China in order to comprehensively understand its quality composition. Subsequently, we employed HS-SPME/HS-GC-MS to deeply analyze the volatile components in litchi honey, with the aim of identifying the key compounds that determine its distinctive aroma, thus accurately delineating the aroma profile of litchi honey.

## 2. Materials and Methods

### 2.1. Honey Sampling

Litchi honey samples were primarily selected from four provinces in southern China, with detailed information provided in Table 1. To ensure the precision and credibility of the experimental results, we carefully selected a large number of pure, untreated litchi honey samples, all provided by experienced beekeepers. The selected honey samples were finely filtered to remove all impurities. After collection, the honey samples were properly stored in a low-temperature freezer at −18 °C to maintain their quality and prepare for subsequent comprehensive analysis. Before use, the samples were thoroughly stirred with a clean, sterile glass rod to ensure uniformity. The plant source of the honey can be determined by analyzing the pollen grains in the samples. In determining the type of honey, in addition to pollen analysis, we also considered its sensory characteristics, harvest time, and geographical origin.

### 2.2. Reagents

Chromatographic grade acetonitrile and methanol were purchased from Merck Company. High-purity (≥99%) standards of fructose, glucose, sucrose, maltose, and hydroxymethylfurfural (HMF) were provided by Shanghai Aladdin Bio-Chem Technology Co., LTD, Shanghai, China. The reagents used in the experiment, including iodine, potassium iodide, sodium acetate, glacial acetic acid, sodium chloride, and soluble starch, were of analytical purity and were purchased from China National Pharmaceutical Group Chemical Reagent Co., Ltd. Shanghai, China. The olive oil isotope standard (ALPHA) was supplied by Shanghai yuanye Bio-Technology Co., Ltd., and sodium tungstate was provided by Sinopharm Chemical Reagent Co., Ltd., Shanghai, China.

### 2.3. Melissopalynological Analysis

The plant source of honey was determined by analyzing the composition of pollen grains in honey samples. A 10 g sample of honey was dissolved in 20 mL of distilled water at 20–40 °C, placed in a centrifuge tube, and centrifuged at 1000× *g*-force for 10 min, after which the supernatant was discarded. Another 20 mL of distilled water was added to completely dissolve the remaining sugar crystals and centrifuged again at 1000× *g*-force for 5 min to completely remove the supernatant. A sterile microspatula was used to spread the sediment evenly on a microscope slide, and the sample was allowed to dry for a period of time. Subsequently, a drop of glycerin was added to the cover slip, and a pollen reference atlas was used to identify the morphology of pollen grains in the honey. The percentage of pollen types in each honey sample was calculated based on the total number of different types of pollen grains in each sample. If the relative frequency of pollen from a taxon exceeded 45%, the nectar was classified as monofloral honey. Pollen counting was conducted using an Olympus corporation optical microscope, Tokyo, Japan, connected to a computer.

### 2.4. Determination of Physicochemical Indicators

Quality testing of honey is a delicate and complex process that encompasses multiple key physicochemical properties. These properties included the precise determination of moisture content, detailed analysis of HMF concentration, in-depth assessment of electrical conductivity, accurate calculation of the ratio of reducing sugars to disaccharides, and rigorous detection of C-4 sugar adulteration. The comprehensive consideration of these indicators formed an important line of defense in ensuring the purity and overall quality of honey.

#### 2.4.1. Sensory Inspection Method

According to the standards of the SN/T 0852-2012 [17], honey should exhibit its characteristic color, smell, and taste.

Color Inspection: The honey sample was poured into the Hanna Honey Colorimeter for color comparison to read the color value and determine its color. Based on the depth of color, honey could be categorized into the following types: water white (below 8 mm), extra white (below 16 mm), white (below 34 mm), extra light amber (below 50 mm), light amber (below 85 mm), amber (below 114 mm), and dark (below 140 mm).

Smell and Taste Inspection: A clean glass rod was used to stir the sample to sniff its smell; then, using the same glass rod, the honey was lifted to taste its flavor.

#### 2.4.2. Measurement of Moisture Content

The moisture content had been determined in accordance with SN/T 0852-2012 [17]. First, the Abbé refractometer was connected to the ultra-thermostat, and the temperature of the ultra-thermostat was set to 40 °C. Before measurement, the refractive index of the refractometer had been calibrated with fresh distilled water. The prism was thoroughly cleaned before measurement to avoid any residual substances that might have affected the accuracy of the determination. A glass rod was used to dip 1~2 drops of the well-mixed sample, which were then dropped onto the lower prism. The prism was quickly closed and allowed to stand for a few seconds to let the sample reach 40 °C. The alignment with the light source was made, and the boundary between light and dark was observed through the eyepiece. The compensator screw was rotated to make the boundary clear, and the scale pointer screw was adjusted so that the boundary between light and dark passed exactly through the intersection of the crosshairs on the objective lens. The refractive index was then read on the scale, ensuring that the temperature was exactly 40 °C. The results were calculated as follows:

X1 = 100 − [78 + 390.7(n − 1.4768)](1)

where: X1 represented the moisture content in the sample, %. n was the refractive index of the sample at 40 degrees.

#### 2.4.3. Carbohydrate Analysis Method

The measurement of carbohydrates in honey, including fructose, glucose, sucrose, and maltose, was conducted in accordance with the GB/T 18932.16-2003 [18].

Aliquoted 1~2 g of well-mixed honey sample into a 100 mL colorimetric tube, approximately 50 mL of water was added, and the mixture was vortexed until fully dissolved. The solution was then transferred to a 100 mL volumetric flask and diluted to the mark with water, followed by thorough mixing. After centrifugation to obtain the supernatant, the solution was filtered through a 0.45 μm aqueous filter membrane into a sample vial for HPLC analysis. Quantification was performed using an external standard method. The analysis was performed using the Shimadzu HPLC-20ADXR system by Shimadzu Corporation, Kyoto, Japan, equipped with an amino column (4.6 mm × 250 mm, particle size 5 μm). The mobile phase consisted of acetonitrile and water in a volume ratio of 70:30, with a flow rate of 1.0 mL/min. The column temperature was maintained at 40 °C, and the injection volume was 10 μL.

#### 2.4.4. Determination of Amylase Activity

This study adhered to the GB/T 18932.16-2003 [18] for the determination of amylase activity in honey. The assay principle involved the addition of a starch solution to the honey sample solution, where the amylase present in the honey had hydrolyzed a portion of the starch. The remaining starch reacted with added iodine to produce a blue-purple color, which faded as the reaction progressed. The time required to reach a specific absorbance at a wavelength of 660 nm was measured using a spectrophotometer, allowing for the calculation of the volume of starch hydrolyzed per gram of honey in one hour.

The preparation of the iodine stock solution involved weighing 8.8 g of iodine and dissolving it in 30 mL of water containing 22 g of potassium iodide, followed by dilution to 1000 mL. The iodine solution was prepared by dissolving 20 g of potassium iodide, adding 5.0 mL of the iodine stock solution, and diluting it to 500 mL, with a new batch prepared every two days. The acetate buffer (pH 5.3) was prepared by dissolving 87 g of sodium acetate in 400 mL of water, adding 10.5 mL of glacial acetic acid, and diluting to 500 mL, adjusting the pH to 5.3 with sodium acetate or glacial acetic acid as needed. The starch solution was prepared by dissolving 2.000 g of soluble starch in 90 mL of water, boiling and simmering for 3 min, cooling to room temperature, and then transferring to a 100 mL volumetric flask and diluting to the mark.

Weighed 5 g of the sample into a 20 mL beaker, added 15 mL of water and 2.5 mL of acetate buffer, and transferred to a 25 mL volumetric flask containing 1.5 mL of sodium chloride solution, then diluted to the mark. Took 5.0 mL of the starch solution, 10.0 mL of the sample solution, and 10.0 mL of the iodine solution and preheated them in a 40 °C water bath for 15 min. Pour the starch solution into the sample solution, mix thoroughly by tilting back and forth, and start timing. After 5 min, take 1.0 mL of the mixed sample solution and add it to 10.0 mL of the iodine solution, diluted with the volume of dilution water determined by the starch solution calibration, and mix thoroughly. Measured the absorbance at 660 nm using Shimadzu UV2700i-VIS by Shimadzu Corporation, Kyoto, Japan and a constant temperature water bath.

On logarithmic graph paper, plot the absorbance (%) on the *y*-axis and time (min) on the *x*-axis, marking the measured absorbance values and their corresponding times. Connected the points to draw a straight line. From this line, determined the corresponding time at which the absorbance of the sample solution intersected with 0.235. The result was calculated using the formula:

X = 300/t(2)

where X is the amylase value in the sample solution, expressed in mL per g per h [mL/(g h)], and t is the corresponding time in min (min).

#### 2.4.5. Conductivity Measurement

This study adhered to the GB/T 18932.15-2003 [19] for the measurement of conductivity in honey. The measurement principle involved diluting a sample equivalent to 20 g of anhydrous honey to a final volume of 100 mL with water, followed by the determination of its conductivity at 20 °C using the FE38 Basic Conductivity Meter by Mettler Toledo Zurich.

The mass of the honey sample equivalent to 20 g of anhydrous honey was calculated using the formula:

m = 20/(1 − c)(3)

where m represented the mass of the honey sample in g, and c denoted the moisture content of the honey sample as a percentage.

The sample was weighed according to the calculated mass and transferred to a 100 mL beaker. The water added was about 40 mL and was stirred with a glass rod until complete dissolution, and the solution was transferred to a 100 mL volumetric flask. The beaker was washed with 30 mL of water in several portions, and the washings were transferred to the volumetric flask. Finally, it was diluted to the mark with water and mixed thoroughly.

The prepared sample solution was poured into a 100 mL beaker, and the conductivity cell was inserted into the solution, gently agitating to dislodge any bubbles. The conductivity of the sample solution was recorded once the reading stabilized, with results reported in mS/cm.

#### 2.4.6. Isotope Detection

In adherence to the GB/T 18932.1-2002 [20] standard, this experiment employed a state-of-the-art online, fully automated Dumas combustion tube technique. This method involved the combustion of the sample under controlled conditions of pure oxygen pulses and the presence of a catalyst. Subsequent to combustion, the resulting products were subjected to rigorous chemical purification and separation via gas chromatography (GC). Following this preparation, the δ13C values were accurately quantified using a sophisticated Integra-2 stable carbon isotope mass spectrometer (EA-IRMS). The δ13C value obtained from honey protein served as a benchmark for comparison with the δ13C value of the honey itself. The discrepancy between these two values was utilized to determine the concentration of carbon-4 plant sugars within the honey sample.

Preparation of Honey Protein Samples Commenced by accurately weighing 10.0 g to 2.0 g of pristine honey samples into a 50 mL centrifuge tube. Four milliliters of water was added and mixed thoroughly. Furthermore, a combination of 2.0 mL of a 10% sodium tungstate solution with 2.0 mL of 0.335 mol/L sulfuric acid solution was added in a 20 mL measuring cylinder, mixing rapidly before transferring the mixture to the centrifuge tube containing the honey sample, and a re-mixed the contents was completed. Subsequently, the mixture was incubated in an 80 °C water bath for a minimum of 30 min, intermittently rotating the centrifuge tube’s contents for 20 s every 5 to 10 min until a flocculent precipitate was formed. Then, the centrifuge tube was filled with water, mixed, and centrifuged at 1500× *g* for 5 min, discarding the supernatant. Afterward, the precipitate was washed thoroughly with approximately 50 mL of water and re-centrifuged, repeating this washing process five times. Ultimately, the supernatant was discarded, and the centrifuge tube was transferred with the precipitated protein to a 75 °C oven for drying, exceeding 3 h. Once dried, weighed out 3.0 mg of protein and transferred it to an 8 mm× 5 mm tin cup, ensuring it was sealed and stored, with duplicates prepared for each sample.

For the standard and control samples, pipetted 2 µL of olive oil standard into an 8 mm × 5 mm tin cup, sealed it, and reserved it as the reference for the honey sample test batch. In addition, took 1 µL of olive oil standard and injected it into another 8 mm × 5 mm tin cup, sealed it, and preserved it as the standard and control for the honey protein sample test batch.

Organized the samples to be tested on the automatic sampler in accordance with the pre-established test batch sequence. Executed the test batch under precise computer control. Following the completion of measurements, the δ13C value of the sample was automatically computed by the system. It was imperative that the δ13C values for both honey and its protein were measured under identical instrumental conditions.

The results were calculated as follows:

(4) $$X(\%)=\frac{\delta 13\mathrm{Cp}-\delta 13\mathrm{CH}}{\delta 13\mathrm{Cp}-(-9.7)}\times 100$$

In the formula: X—represented the percentage content of C-4 plant sugars in honey; δ13Cp—represented the δ13 value of proteins in honey; δ13CH—represented the δ13 value of honey.

#### 2.4.7. Determination of HMF Content

The determination of HMF content in this study was based on the GB/T 18932.18 [21]. An Agilent 1200 Series from Agilent Technologies, Inc., Santa Clara, CA, USA, with an Agilent C18 column (5 μm, 250 mm × 4.6 mm) was used. The mobile phase was a mixture of methanol and water (volume ratio 10:90), with the flow rate set at 1.0 mL/min. The detection wavelength was 285 nm, the column temperature was maintained at 30 °C, and the injection volume was 10 μL.

Accurately weighed 10.00 g of the sample and placed it in a 100 mL beaker, added 10 mL of methanol, and gently stirred with a glass rod to ensure complete dissolution of the sample. Subsequently, the solution was transferred into a 100 mL volumetric flask, diluted to the mark with water, and mixed thoroughly. Finally, the solution was filtered through a 0.45 μm filter membrane for subsequent analysis.

### 2.5. Volatile Substances Analysis

#### 2.5.1. Extraction of Volatile Substances

To precisely extract the volatile components from litchi honey, this study employed the HS-SPME technique and conducted an in-depth analysis on samples 2, 3, 4, and 6 in Table 1. During the extraction process, we specifically selected the Supelco 57328-U 50/30 DVB/CAR/PDMS solid-phase extraction fiber(Supelco, Inc., Darmstadt, Germany), to ensure efficient and reliable capture of the components.

The following is an elaborate protocol that had been meticulously followed for the extraction of volatile constituents from the honey specimens: Weighed 1.5 g of honey sample was placed in an 8 mL headspace vial, 0.3 mL of deionized water was added, and a magnetic stirrer bead was used to mix thoroughly, then the vial was balanced in a 70 °C water bath for 10 min before inserting the SPME extraction fiber for extraction for 50 min. After extraction, the fiber was removed and quickly inserted into the GC-MS injection port for desorption at 230 °C for 5 min. Before sample extraction, the extraction fiber was regenerated at 250 °C for 5 min.

#### 2.5.2. GC-MS Conditions

A 7890B-7000C Gas Chromatograph-Mass Spectrometer, Chromatographic Column: 30 m × 0.25 mm × 0.25 μm (both from Agilent Technologies, Inc., CA, USA), with an inlet temperature of 250 °C. The sample was injected at a constant flow rate of 1 mL/min (helium) without split. The temperature program started at 40 °C, increased to 160 °C at a rate of 4 °C/min and held for 3 min, then increased to 280 °C at a rate of 10 °C/min and held for 3 min. The mass spectrometer detector was set at a temperature of 250 °C, with a scan range of *m*/*z* 30–550.

#### 2.5.3. Qualitative Analysis of Volatile Substances

The volatile compounds were identified by matching with the National Institute of Standards and Technology (NIST 17) mass spectral database and retention indices (RI, determined by C7-C40 n-alkanes). The Threshold of Similarity for mass spectral matching was established to be greater than 80%.

## 3. Results and Discussion

### 3.1. Characterization of Pollen Types in Honey

**Figure 1:**
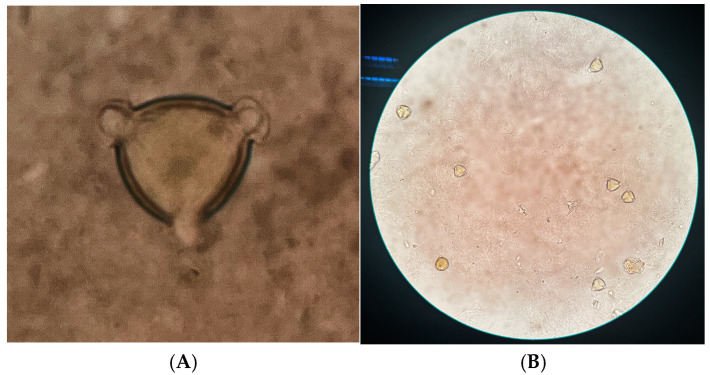
Pollen grain morphology identified from honey samples. (**A**) Standard microscopic image of litchi pollen, (**B**) Microscopic image of honey sample.

The relative frequency of litchi nectar plants and the morphology of pollen grains are shown in Table 1 and Figure 1, respectively. All six samples were considered litchi honey because the litchi pollen count in each honey sample was higher than 45%.

### 3.2. Analysis of Basic Physicochemical Indicators

#### 3.2.1. Sensory Data and Interpretations

As indicated in Table 2, sample No. 4 stood out as white (below 34 mm), while the remaining five samples fell into the category of extra light amber (below 50 mm). Across the spectrum from white to extra light amber, all six honey samples exuded a delightful litchi aroma and exhibited a silky-smooth consistency.

#### 3.2.2. Moisture Content

Moisture content stood as a pivotal metric in assessing the quality of honey. This content was influenced by a multitude of factors, encompassing the variety of bees, the types of nectar-producing plants, the honey-making process, as well as the timing of honey collection, and geographical variations. In the honey-making process, bees effectively remove excess moisture from the nectar by energetically vibrating their wings. An excessive moisture level in honey could predispose it to fermentation, which in turn could compromise its flavor and overall quality.

Usually, the water content of mature honey is below 20%. According to the data in Table 2, the moisture content of unprocessed litchi honey had ranged from 17.18% to 22.7%. This indicated that the moisture content of mature litchi honey was relatively high. As indicated in Table 2, the moisture content of the six litchi honey samples examined in this research all fell below the 23% threshold, thus fulfilling the criteria for grade one litchi honey, which stipulates a moisture content of less than 23%, as outlined in the reference [22]. The somewhat elevated moisture content in litchi honey could be primarily attributed to the warm and humid conditions that characterized the growing environment of litchi, a tropical fruit, leading to naturally higher moisture content in litchi nectar. Furthermore, the honey production period at the tail end of spring and the start of summer, marked by increased rainfall and high atmospheric humidity, also played a role in the elevated moisture levels within the honey. In line with the guidelines set by the AOAC [23] and CAC [24], while the moisture content of heather honey was permitted to reach up to 23%, other types of honey should not have exceeded 20%. Consequently, it is advisable to set the moisture content requirement for litchi honey at no more than 23%.

#### 3.2.3. Conductivity

Conductivity was a pivotal procedure in the quality control of honey, serving to evaluate its purity, geographical origin, floral type, maturity, and moisture content, as well as to monitor the processing sequence to ensure food safety and quality. According to CAC regulations, the conductivity of honey should not exceed 0.8 mS/cm. The GB/T 18932.15-2003 [19] had been formulated by referencing and refining the CAC guidelines, demonstrating a strong alignment with international standards in the measurement of honey conductivity. As indicated in Table 2, the conductivity of all six honey samples was not higher than 0.28 mS/cm, which meets the standard requirements.

#### 3.2.4. Amylase Value

The enzyme activity in honey was a key indicator for assessing its quality and texture, playing a decisive role in the process of honey production, driving the transformation from nectar to honey. According to the regulations of CAC, the amylase activity in processed honey must not be less than eight units, while honey with lower natural enzyme activity should not fall below three units to ensure the freshness and maturity of honey and maintain uniform quality and safety standards for honey products in the international market. According to reference [22], the amylase activity of litchi honey should not be less than 2 mL/(g·h). After testing, all six samples of litchi honey exceeded 7.7 mL/(g·h), reflecting their high-quality characteristics.

#### 3.2.5. Quantitative Analysis of Carbohydrate Composition

Reducing sugars held a dominant position in honey, primarily composed of fructose and glucose. The ratio of these two sugars was the core criterion for assessing the quality of honey. The content of fructose and glucose not only directly affected the taste and quality evaluation of honey but was also a crucial factor in determining its hygroscopic and rheological properties. In accordance with the guidelines set by the AOAC and the CAC, the combined concentration of glucose and fructose in honey was required to be over 60%, while the sucrose content must not have surpassed 10%. This standard mirrored the natural transformation that occurred when bees, using their enzymes, converted the sucrose present in nectar into glucose and fructose. An elevated sucrose level might have suggested that the honey was either immature or had been tampered with. GB14963-2011 and reference [22] also upheld this rigorous criterion. According to the data presented in Table 2, the fructose content in litchi honey ranged from 36.5% to 39.6%; the glucose content, on the other hand, ranged from 30.57% to 37.63%. Overall, the total content of glucose and fructose was between 69.63% and 77.23%. The sucrose content in litchi honey ranged from 0.59% to 1.15%. The combined fructose and glucose levels, along with the sucrose content in all six litchi honey samples, aligned perfectly with the prescribed standards.

The ratio of fructose to glucose (F/G) in honey was a crucial factor in determining its crystallization rate [25]. The contribution of botanical origin and sugar composition of honey to the crystallization phenomenon was noted. Due to the relatively low solubility of glucose, when the F/G ratio exceeds 1.33, the honey was less likely to crystallize over an extended period; conversely, when the F/G ratio was below 1.11, the honey tended to crystallize quickly. According to the data in Table 2, the F/G ratio of litchi honey ranged from 1.05 to 1.28, with an average of 1.12. This indicated that as the storage time of litchi honey increased, it became more prone to crystallization.

The ratio of fructose to reducing sugars in litchi honey ranged from 51.28% to 56.22%, highlighting the abundance of fructose, which dominated the total reducing sugar content, thereby endowing the honey with a richer and more mellow sweetness. The stability of this ratio reflected the reliability of the quality of litchi honey and served as a key indicator for its quality assessment. Moreover, this unique ratio also revealed the distinctive floral source attributes of litchi honey, making it an excellent choice for consumers who prefer a sweet taste in their honey.

The content of maltose in honey served as a crucial indicator of its quality and maturity. A low maltose level typically signified that the honey was mature and of high quality, whereas a high content suggested that the honey was not fully mature, had been adulterated, or improperly processed, and it could also have reflected different plant sources. The European Union stipulates that the maltose content in honey must not exceed 5%. As indicated in Table 2, the maltose content in all six litchi honey samples ranged from 1.09% to 1.51%.

#### 3.2.6. HMF

HMF was a compound produced during the heating of sugars, and its high levels might have indicated that honey had been overheated or improperly stored, affecting the quality of the honey. As per the guidelines set by CAC, the European Union, and reference [22], HMF concentrations at or above 40 mg/kg (or 80 mg/kg in tropical regions) were deemed detrimental to human health. The data in Table 2 indicated that the HMF content across the six honey samples varied between 1.04% and 3.49%, which was considered relatively low and aligned with the established quality criteria.

#### 3.2.7. The Stable Carbon Isotope Ratio Analysis

The Stable Carbon Isotope Ratio Analysis (SCIRA) was a method promoted by AOAC in 1998. GB/T 18932.1-2002 [20] adopts the technology of combining an Elemental Analyzer with an EA-IRMS. This technique was widely utilized in the authentication of honey [26,27] and the tracing of its geographical origin [28,29].

The δ13C signature in honey, a measure of carbon isotope ratios, was instrumental in pinpointing the origin of honey by disclosing the ratio of C-13 to C-12. Protein δ13C values offered insights into the bees’ dietary sources, while the δ13C values of honey, glucose, and fructose mirrored the plant origin, environmental conditions, and detailed information about the nectar-producing flora. The δ13C values of disaccharides in honey related to the plant source of the residual disaccharides collectively serve as a scientific foundation for honey’s authenticity and quality assurance. The protein δ13C values for the six honey samples predominantly fell within the −26‰ to −24‰ range, signifying a uniform carbon isotope profile that hinted at a shared origin from comparable environmental settings or plant varieties. The honey δ13C values from the samples exhibited a high degree of uniformity in carbon isotope traits, with values generally clustering around −25‰, implying a likely common environmental backdrop or plant type. The δ13C values for glucose and fructose were both clustered around −25.5‰, indicating that these honey samples may have shared a similar plant origin or growing environment. The δ13C values of disaccharides across the six honey samples spanned from −25.44‰ to −27.17‰, uncovering an intricate mosaic of plant sources and environmental influences. The average δ13C value of −26.94‰ signified a lighter carbon isotope footprint, commonly associated with C3 plants and shaped by surrounding environmental conditions. This was consistent with litchi being a C3 plant.

Evaluating this data set, the δ13C indicators of the honey samples, despite showcasing some variability, predominantly suggested a shared nectar source, facilitating the authentication and quality monitoring of the honey. The research conducted by Simsek et al. [24], which utilized EA-IRMS to ascertain the δ13C values of authentic honey and protein, had reported ranges of −23.30‰ to −27.58‰ and −24.13‰ to −26.76‰, respectively, corroborating the spectrum of our analyzed values.

According to the calculation Equation (4), if the calculation result was negative, the content of C-4 plant sugars was considered as 0; if the calculation result was greater than or equal to 7%, it should have been deemed to contain C-4 plant sugars (primarily referring to corn sugar or sucrose). Based on the results in Table 2, none of the six honey samples contained C-4 plant sugars.

### 3.3. Analysis of Volatile Components

**Figure 2:**
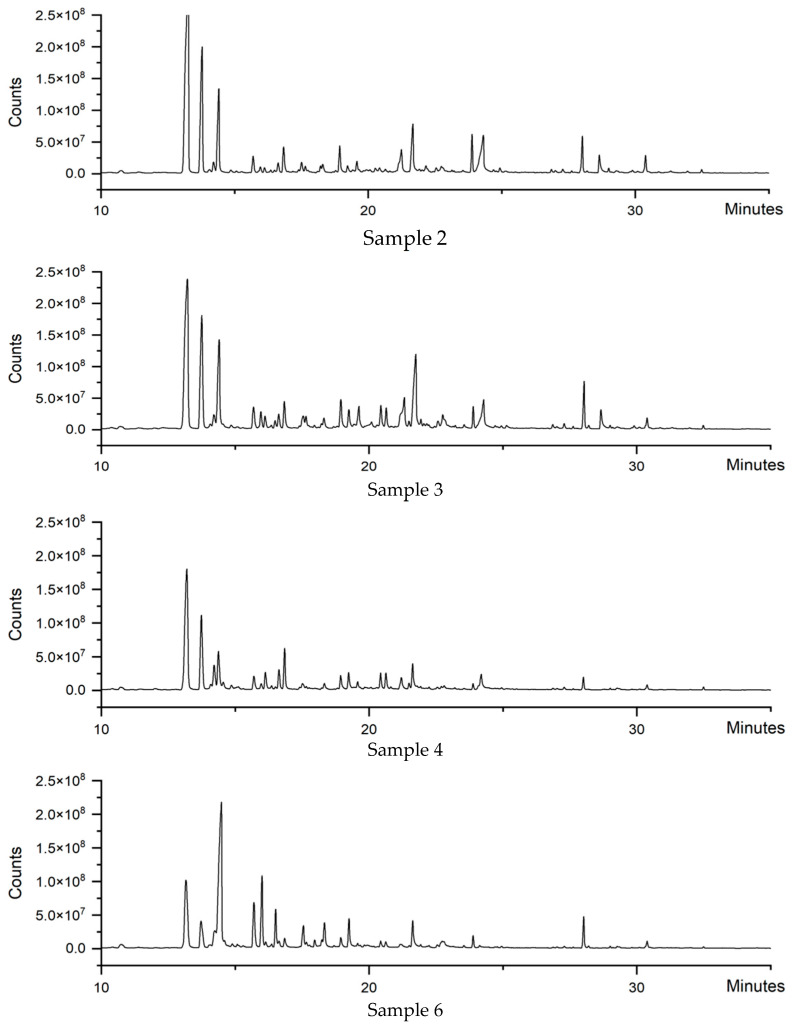
Total ion chromatogram of volatile components in four honey samples.

Volatile compounds were integral to the distinctive aroma of honey, with over 600 such components having been identified to date, encompassing aldehydes, alcohols, ketones, acids, esters, alkanes, and phenols, among others [25]. The variability in volatile constituents across different honey sources imparted each with a unique flavor profile. The presence of identical volatile compounds and/or their metabolites in the same type of nectar, flower, and honey could serve as biomarkers for the floral origin of honey [26]. This study employed HS-SPME-GC-QQQ to analyze the volatile constituents of litchi honey, with the total ion chromatogram depicted in Figure 2. A total of 37 volatile components were identified through NIST database matching and retention index comparison, as detailed in Table 3. The four honey samples exhibited 26 shared volatile components, predominantly featuring terpenes, alcohols, aldehydes, and aromatic compounds, in line with the findings reference [30]. Notably, trans-linalool oxide, linalool, lilac aldehyde B, lilac aldehyde D, α-terpineol, and cedrol stand out as the distinctive volatile signatures of litchi honey. These components are crucial for the quality assessment and selection of litchi honey, revealing its plant origin and reinforcing its connection with litchi flowers, thus enhancing the sensory experience. The volatile compounds determine the aromatic quality of the honey, setting litchi honey apart in the market. In particular, linalool, with its unique litchi flower fragrance, intensifies the honey’s distinctive flavor, boosts its market competitiveness, and solidifies its reputation for high quality.

It is noteworthy that litchi honey has been found to contain high abundances of hexadecamethylcyclooctasiloxane, octamethylcyclotetrasiloxane, decamethylcyclopentasiloxane, and dodecamethylcyclohexasiloxane. These methylcyclosiloxanes are a type of organosilicon compound, with octamethylcyclotetrasiloxane, decamethylcyclopentasiloxane, and dodecamethylcyclohexasiloxane being common varieties. As multifunctional industrial chemicals, they are primarily used as lubricants, softeners, defoamers, and sealants. The detection of these compounds in honey may indicate environmental pollution or the use of silane-based additives in agricultural activities. The presence of methylcyclosiloxanes in honey raises dual concerns about food safety and environmental protection, particularly considering their persistence, potential for bioaccumulation, and the risks they may pose to the ecology and health. Although their concentrations in honey are typically low, their detection highlights the necessity of thoroughly investigating the sources, pathways of dissemination, and potential exposure risks during the production and processing of honey.

### 3.4. Variations in Honey Quality Among Bee Species

Based on the data analysis of Table 2 and Table 3, although the honey samples collected by *Apis mellifera* and *Apis cerana* bees may have exhibited certain differences in physicochemical properties and volatile components, statistically, no systematic significant differences had been found between the honey produced by the two bee species. Behind this phenomenon, it is likely that the physicochemical indicators and volatile compounds in honey had primarily been inherited from plant nectar, thus revealing a key point: the characteristic quality of the floral source had had a decisive impact on the final composition of honey.

## 4. Conclusions

This study conducted an in-depth and comprehensive analysis of the basic quality indicators and volatile components in six litchi honey samples. The results showed that all samples met the relevant quality control requirements for honey set by both Chinese and CAC, indicating that the honey raw materials provided by beekeepers in the market have reached the qualified quality standards.

This study conducted an in-depth analysis of the volatile components in four litchi honey samples, identifying key volatiles such as trans-linalool oxide, linalool, eugenol B, eugenol D, α-terpineol, and cedrol, which are crucial in shaping the unique flavor and aroma characteristics of litchi honey.

In this study, the tested parameters of honey samples collected from *Apis mellifera* and *Apis cerana* revealed no statistically significant systematic differences, highlighting the profound influence that floral source characteristics have on shaping the composition of honey.

It is worth noting that the detection of methyl cyclosiloxane in litchi honey has raised concerns about food safety and environmental issues. Although the concentration in honey is low, the persistence and potential for bioaccumulation urgently require an in-depth investigation into the production process of honey.

## Figures and Tables

**Table 1:** Specification of honey samples.

Number	Honeybee Species	Geographical Origin	Plant Species	Harvest Time	Pollen Grain Counted (%)
1	*Apis* *mellifera*	Guangdong province	Litchi chinensis ’Nuo Mi Ci’	April 2022	61.25
2	*Apis cerana*	Guangdong province	Litchi chinensis ’Nuo Mi Ci’	April 2022	71.01
3	*Apis cerana*	Fujian province	Litchi chinensis ’Chen Zi’	May 2022	72.88
4	*Apis cerana*	Hainan province	Litchi chinensis ’Feizixiao’	March 2022	53.87
5	*Apis cerana*	Guangxi province	Litchi chinensis ’Gui Wei’	April 2022	82.38
6	*Apis mellifera*	Guangxi province	Litchi chinensis ’Gui Wei’	April 2022	76.79

**Table 2:** Physico-chemical analysis of litchi honey samples.

Parameter	Sample Code					
	1	2	3	4	5	6
Moisture content/%	17.18 ± 0.06 E	20.09 ± 0.16 D	22.13 ± 0.10 B	22.70 ± 0.08 A	21.66 ± 0.16 C	22.00 ± 0.14 B
Color/mm	46.0 ± 1.0 A	47.3 ± 0.6 A	35.3 ± 0.6 B	31.0 ± 1.0 C	35.7 ± 2.1 B	33.7 ± 0.6 BC
Fructose/%	39.60 ± 0.25 A	38.41 ± 0.27 ABC	39.25 ± 0.27 AB	37.46 ± 0.34 ABC	36.66 ± 2.22 BC	36.50 ± 0.46 C
Glucose/%	37.63 ± 0.35 A	36.29 ± 0.47 AB	30.57 ± 0.42 D	34.01 ± 0.62 BC	32.97 ± 2.01 CD	33.14 ± 0.14 C
Sucrose/%	1.15 ± 0.06 A	0.72 ± 0.03 C	1.11 ± 0.08 A	0.59 ± 0.01 D	0.97 ± 0.03 B	0.90 ± 0.02 B
Maltose/%	1.44 ± 0.18 A	1.09 ± 0.03 B	1.51 ± 0.08 A	1.32 ± 0.04 AB	1.35 ± 0.09 A	1.38 ± 0.05 A
Reducing Sugar/%	77.23 ± 0.57 A	74.70 ± 0.69 AB	69.83 ± 0.68 BC	71.47 ± 0.94 BC	69.63 ± 4.22 C	69.64 ± 0.55 C
Fructose/Reducing Sugar/%	51.28 ± 0.13 C	51.43 ± 0.23 C	56.22 ± 0.18 A	52.41 ± 0.27 B	52.65 ± 0.16 B	52.42 ± 0.27 B
F/G	1.05 ± 0.01 C	1.06 ± 0.01 C	1.28 ± 0.01 A	1.10 ± 0.01 B	1.11 ± 0.01 B	1.10 ± 0.01 B
HMF/mg/kg	1.04 ± 0.05E	1.33 ± 0.09 DE	3.49 ± 0.13 A	2.75 ± 0.16 B	1.65 ± 0.03 CD	1.79 ± 0.19 C
Amylase value/mL/g·h	14.09 ± 0.53 A	8.22 ± 0.76 BC	7.75 ± 0.52 C	7.72 ± 0.60 C	8.07 ± 0.68 BC	9.60 ± 0.65 B
Conductivity mS/cm	0.21 ± 0.01 C	0.25 ± 0.01 B	0.28 ± 0.01 A	0.21 ± 0.01 C	0.16 ± 0.01 D	0.15 ± 0.01 D
Protein δ13C‰	−24.28 ± 0.14 A	−24.36 ± 0.09 A	−25.20 ± 0.06 B	−25.66 ± 0.06 C	−24.95 ± 0.13 B	−24.97 ± 0.13 B
Honey δ13C‰	−25.53 ± 0.09 B	−25.51 ± 0.18 B	−25.19 ± 0.04 A	−25.96 ± 0.12 C	−25.64 ± 0.05 B	−25.51 ± 0.07 B
Glucose δ13C	−25.28 ± 0.29 A	−25.52 ± 0.28 AB	−25.24 ± 0.13 A	−25.88 ± 0.08 B	−25.55 ± 0.20 AB	−25.35 ± 0.16 AB
Fructose δ13C	−25.75 ± 0.13 BC	−25.52 ± 0.09 AB	−25.24 ± 0.19 A	−26.08 ± 0.22 C	−25.71 ± 0.23 BC	−25.64 ± 0.09 ABC
Disaccharide δ13C	−26.10 ± 0.33 AB	−26.51 ± 0.23 BC	−25.44 ± 0.22 A	−27.17 ± 0.43 C	−27.19 ± 0.18 C	−27.17 ± 0.09 C
Disaccharide/%	3.28 ± 0.07 A	2.28 ± 0.05 C	2.92 ± 0.14 B	2.39 ± 0.09 C	3.53 ± 0.05 A	3.41 ± 0.13 A
Trisaccharide/%	ND	ND	ND	ND	ND	ND
Oligosaccharide/%	ND	ND	ND	ND	ND	ND

The values in the table indicate the mean ± standard deviation of three repetitions. The uppercase letters within the same parameter group exhibited a significant difference at a level of 0.05.

**Table 3:** Volatile Components in litchi Honey.

RT(min)	Chemical Compound	Confidence(%)	Molecular Formula	CAS	Peak Area (×10^6^)			
					1	2	5	6
9.6569	1-heptanol	91.8	C_7_H_16_O	111-70-6	20.53			
10.7005	octamethyl-cyclotetrasiloxane	93.3	C_8_H_24_O_4_Si_4_	556-67-2	0.80	14.43	14.51	14.00
11.9884	3,5,5-trimethyl-3-cyclohexen-1-one	90.4	C_9_H_14_O	471-01-2	0.11		0.14	0.05
13.1558	trans-Linalool oxide	96.9	C_10_H_18_O_2_	34995-77-2	249.92	33.59	234.66	133.27
13.2223	Ethyl-2-(5-methyl-5-vinyltetrahydrofuran-2-yl)propan-2-yl carbonate	97.9	C_13_H_22_O_4_	1000373-80-3	107.93	111.29	111.42	45.79
14.1985	Linalool	96.3	C_10_H_18_O	78-70-6	9.91	14.28	28.11	27.49
14.4065	3,7-dimethyl-1,5,7-octatrien-3-ol	94.8	C_10_H_16_O	29957-43-5	142.91	150.82	49.92	368.06
14.5553	Isophorone	88.9	C_9_H_14_O	78-59-1	7.47	8.18		7.78
15.1222	tetrahydro-4-methyl-2-(2-methyl-1-propenyl)-2H-pyran	91.7	C_10_H_18_O	16409-43-1			0.84	2.12
15.2483	1,3,8-p-Menthatriene	90.8	C_10_H_14_	18368-95-1	0.96			0.08
15.6926	2,6,6-Trimethyl-2-cyclohexene-1,4-dione	92.2	C_9_H_12_O_2_	1125-21-9	26.34	36.36	23.44	27.81
15.9586	Lilac aldehyde B	95.2	C_10_H_16_O_2_	53447-45-3	5.38	16.3	2.43	62.85
16.1124	3,6-dihydro-4-methyl-2-(2-methyl-1-propenyl)-2H-Pyran	96	C_10_H_16_O	1786-08-9	6.28	18.21	20.05	6.41
16.3478	Cyclopentasiloxane, decamethyl-	98.1	C_10_H_30_O_5_Si_5_	541-02-6	2.13	3.21	3.34	2.26
16.488	Lilac aldehyde D	95.2	C_10_H_16_O_2_	53447-47-5	3.26	8.26	2.45	34.06
16.8330	(3R,6S)-2,2,6-Trimethyl-6-vinyltetrahydro-2H-pyran-3-ol	97.1	C_10_H_18_O_2_	39028-58-5	25.67	16.09	43.00	7.32
17.3901	α-Terpineol	88.4	C_10_H_18_O	98-55-5	5.48	8.64	6.74	1.86
17.5468	2,6-dimethyl-3,7-Octadiene-2,6-diol	94.8	C_10_H_18_O_2_	13741-21-4	23.14	36.00		49.78
17.6443	2,6,6-trimethyl-1,3-cyclohexadiene-1-carboxaldehyde	98.3	C_10_H_14_O	116-26-7	4.83	9.22	2.62	4.42
17.9552	Decanal	97.1	C_10_H_20_O	112-31-2		1.83	0.15	4.76
18.2195	alpha,4-dimethyl-3-cyclohexene-1-acetaldehyde	92	C_10_H_16_O	29548-14-9	18.67	10.64	3.06	15.97
18.3247	4-Methyleneisophorone	92.9	C_10_H_14_O	20548-00-9	12.84	7.47	5.65	18.33
18.9364	2-Hydroxy-3,5,5-trimethylcyclohex-2-ene-1,4-dione	97.3	C_9_H_12_O_3_	35692-98-9	15.88	20.21	4.78	9.12
20.0034	Diethylene glycol dibutyrate	84.8	C_12_H_22_O_5_	1000458-46-7	1.30	2.41	1.56	0.075
20.6409	Epoxy-linalooloxide	85.8	C_10_H_18_O_3_	1000007-96-5	19.57	3.10	12.89	5.39
22.7249	6-Methyl-5-octen-2-one	80	C_9_H_16_O	24199-46-0				0.82
23.5352	Propanoic acid, 2-methyl-, 3-hydroxy-2,2,4-trimethylpentyl ester	92.9	C_12_H_24_O_3_	77-68-9	1.40	3.03	1.03	1.82
23.8828	(E)-2-Buten-1-one,1-(2,6,6-trimethyl-1,3-cyclohexadien-1-yl)	96.5	C_13_H_18_O	23726-93-4	53.3	6.61	7.92	16.25
24.936	4-(2,4,4-Trimethyl-cyclohexa-1,5-dienyl)-but-3-en-2-one	89.2	C_13_H_18_O	1000187-51-9	0.71			
25.1318	4-Hydroxy-2,6,6-trimethylcyclohex-1-enecarbaldehyde	93.3	C_10_H_16_O_2_	35692-94-5	0.15	0.14	0.12	
27.014	2’,6’-Dimethyl-4’-propoxyacetophenone	90.2	C_13_H_18_O_2_	1000195-98-1	1.87	1.86	1.11	0.57
28.6623	1,2,3,4-Tetramethoxybenzene	90.2	C_10_H_14_O_4_	21450-56-6	42.98	50.05		
29.0038	(E)-Benzene, 4-(2-butenyl)-1,2-dimethyl	87.4	C_12_H_16_	54340-86-2	4.91	3.42	2.80	1.33
29.27	dodecamethyl-Cyclohexasiloxane	80.3	C_12_H_36_O_6_Si_6_	540-97-6	1.55	0.71	0.73	1.35
30.3222	2,2,4-Trimethyl-1,3-pentanediol diisobutyrate	88.4	C_16_H_30_O_4_	6846-50-0	0.36	0.39	3.54	0.20
30.3855	Cedrol	93.8	C_15_H_26_O	77-53-2	6.25	3.04	2.24	2.90
32.4849	hexadecamethyl-Cyclooctasiloxane	87	C_16_H_48_O_8_Si_8_	556-68-3	1.30	3.55	2.80	0.52

## Data Availability

The original contributions presented in this study are included in the article. Further inquiries can be directed to the corresponding authors.

## Abbreviations

The following abbreviations are used in this manuscript:

MDPI	Multidisciplinary Digital Publishing Institute
DOAJ	Directory of open access journals
TLA	Three letter acronym
LD	Linear dichroism
